# Tophus severity grading and surgical treatment: Chinese Medical Doctor Association Multidisciplinary Expert Consensus Statement and Recommendations, 2025

**DOI:** 10.1097/JS9.0000000000003300

**Published:** 2025-09-23

**Authors:** Yunchuan Pan, Shishuai Lin, Nanfang Pan, Shaowen Cheng, Jiangling Yao, Ping Liang, Xueqin Yan, Juan Li, Zunhong Liang, Zhihu Lin, Zhiyang Qiu, Lei Bao, Leiyuan Zhong, Shudian Lin, Feng Zhan, Yang Chen, Bingyan Liu, Shishi Luo, Long Mi, Hengjie Zhu, Guangji Wang, Jianping Lin, Yaolong Chen, Xiaobing Fu, Yuesheng Huang

**Affiliations:** a Hainan Affiliated Hospital of Hainan Medical University (Hainan Province Wound Repair Center, Department of Bone and Joint Surgery, Medical Imaging Center, Department of Rheumatology and Immunology, Nursing and Wound Management), Haikou, China; b Department of Wound Repair, Department of Medical Imaging, Department of Rheumatology and Immunology, The First Affiliated Hospital of Hainan Medical University, Haikou, China; c Evidence Application Center, Affiliated Hospital of Jiangsu University, Zhenjiang, Jiangsu, China; d Evidence-based Medicine Center, School of Basic Medical Sciences, Lanzhou University, Lanzhou, Gansu, China; e Research Center for Tissue Repair and Regeneration Affiliated to PLA General Hospital, Beijing, China; f Institute of Wound Repair and Regeneration Medicine, Southern University of Science and Technology School of Medicine, Shenzhen, China

**Keywords:** consensus, gouty tophi, recommendations, severity grading, surgical treatment

## Abstract

**Background:**

Tophi, a severe complication of advanced gout, developed in approximately 12–35% of patients with gout, with rates as high as 52.56% being reported. Bone and joint damage and chronic refractory wounds caused by gouty tophus present considerable clinical challenges. However, the optimal surgical timing, perioperative management, and treatment approaches for this condition remain unstandardized. Therefore, an expert consensus statement was developed to provide guidance for surgical intervention and improve clinical management of gouty tophus.

**Materials and methods:**

A multidisciplinary expert committee representing relevant clinical specialties was established, and key questions on tophus surgery were compiled. Eleven scientific literature databases were queried to retrieve relevant papers, and a literature quality assessment was performed. Four meetings (nominal group technique) and two Delphi voting rounds were conducted to define tophus erosion grading criteria and establish recommendations. Consensus was considered to have been achieved when at least 85% of panelists indicated agreement. Perspectives from two patients with tophi were incorporated through interviews.

**Results:**

Grading criteria (grades 1–4) for tophus erosion damage were established, and 14 recommendations for surgical treatment (eight strongly recommended and six conditional) were formulated – two on determining tophus erosion severity, four on perioperative management, and eight on surgical indications and treatment.

**Conclusion:**

This study presents the first consensus to introduce standardized criteria for grading tophus severity through multidisciplinary collaboration and offers evidence-based recommendations on optimal surgical timing, perioperative management, and treatment strategies to enhance patient outcomes and guide clinical practice. These recommendations may serve as a foundation for future research and standardization efforts in the surgical management of gouty tophus globally.

## Introduction

Tophi, a severe complication of advanced gout, are crystalline deposits that infiltrate multiple tissues and organs, causing skin ulceration, infection, and osteoarticular destruction, often leading to irreversible functional impairment and deformity^[1,2]^. They typically develop in patients with long-term uncontrolled disease, with risk increasing with age and disease progression^[3]^. Recent epidemiological data reveal a global gout incidence of 0.10% and prevalence of 0.65% in 2021. In China, the incidence and prevalence rates are 0.15% and 0.81%, respectively. Between 1990 and 2021, the incidence rate increased in China and globally by 23.6% and 17.2%, respectively, and the prevalence rate increased in China and globally by 26.3% and 21.8%, respectively. These above-average increases in China indicate an accelerating disease burden^[4]^. However, standardized epidemiological data on tophus incidence and prevalence remain unavailable. Tophi develop in approximately 12–35% of patients with gout^[5]^, with regional Chinese studies documenting rates as high as 52.56%^[6]^. Moreover, they are associated with increased all-cause mortality, particularly in the presence of comorbidities, such as cardiovascular or chronic kidney disease^[7,8]^. Patients experience a poorer quality of life (QoL) than those with chronic leg ulcers^[7]^, thereby presenting substantial challenges in gout management.

Surgical intervention can restore function more rapidly than drug-based approaches and prevent complications in refractory cases^[9]^. Combined surgical and drug treatment strategies are appropriate for critical sites requiring rapid resection of drug-refractory lesions^[10]^. However, treatment outcomes depend on standardized surgical timing, perioperative management, and technique selection^[11]^. In resource-limited settings, inadequate access to training in diagnosing and managing gouty tophi poses a challenge^[12]^. Variability in treatment knowledge among general surgeons, especially in community settings, contributes to inconsistent management and outcomes. Therefore, a standardized surgical treatment framework, reinforced by training and education to ensure uniform execution, is crucial for improving clinical practice.

Despite the established role of surgery in managing gouty tophus, no globally accepted guidelines or standardized assessment systems have been developed. Current guidelines either lack specific surgical recommendations or only mention intervention for functional impairment in vague, nontechnical terms.^[13–15]^ Limited information and minimal surgeon involvement have hindered the development of evidence-based guidelines for standardized interventions to optimize outcomes and reduce complications. Moreover, the absence of sufficient randomized controlled trials (RCTs) limits clinical decision-making between medical and surgical approaches^[16]^. Reported complication rates vary (13–53%), and data on functional recovery remain inconsistent and noncomparable^[17,18]^.

To address these challenges, the Chinese Medical Doctor Association (CMDA) convened a multidisciplinary expert panel to develop this first evidence-based consensus statement. Key objectives include: establishing imaging- and clinical-based tophus erosion grading criteria and formulating perioperative management and surgical technique recommendations via Delphi and nominal group methods to improve clinical outcomes and QoL. This study complies with the TITAN Guidelines on artificial intelligence (AI) use in research and writing^[19]^.

## Materials and methods

### Initial consensus formulation stage

The working group comprised 81 experts in guidance development, evidence assessment, and consensus formulation. Members were selected based on >15 years of clinical experience in relevant specialties (wound repair, plastic surgery, rheumatology, imaging, bone and joint surgery, nursing, and wound management), regional representation, and active involvement in tophus management. The CMDA-nominated panelists were approved by a steering committee comprising an association chairman and two methodologists. Using the nominal group technique and structured voting, the panel generated 11 Population, Intervention, Comparison, and Outcome (PICO) framework questions addressing four key topics: damage grading and diagnosis, indications, treatment outcomes, and tophus surgery techniques (Supplemental Digital Content 1, available at: http://links.lww.com/JS9/F167). Study development was led by a methodologist and conducted in accordance with the RIGHT guidelines (https://www.right-statement.org/right-statement/checklist).

**HIGHLIGHTS**

- This is the first consensus statement on tophus surgery based on evidence from available literature and expert recommendations.
- Grading criteria for tophus erosion damage (grades 1–4) were established to guide clinical assessment and treatment decisions.
- The recommendations emphasize the importance of postoperative follow-up, adherence to urate-lowering therapy, and functional rehabilitation to optimize patient outcomes.
- The grading criteria and recommendations aim to improve the homogeneity and comparability of surgical interventions for gouty tophus.
- Patient values and preferences, along with multidisciplinary expert input, were integrated into the consensus to ensure broad applicability.

### Expansion of search scope and evidence assessment

Eleven databases – Cochrane Library, Medline, Embase, PubMed, Web of Science, CINAHL Ultimate, Science Direct, NLM, China National Knowledge Infrastructure, China Wanfang Medical Network, and China Biomedical Database (Sinomed) – were searched from inception through 31 May 2025 (Supplemental Digital Content 2, available at: http://links.lww.com/JS9/F167). Search syntax, terms, and synonyms were based on the PICO questions. Four researchers independently performed the search, data extraction, and cross-verification; disagreements were resolved through joint discussions or third-party consultations. Gout-related guidelines, consensus statements, and systematic reviews were evaluated to determine whether tophi were addressed. Citations within included papers were manually reviewed to identify additional articles. Subsequently, a literature quality assessment and systematic review were performed to identify resolved and unresolved tophus-related surgical issues (Supplemental Digital Content 3, available at: http://links.lww.com/JS9/F167).

Evidence was graded using the 2009 Oxford Center for Evidence-Based Medicine guidelines^[20]^. Two researchers independently assessed study quality; disagreements were resolved through discussion. For recommendations supported by multiple evidence levels, the one with the highest level was reported (Supplemental Digital Content 4, available at: http://links.lww.com/JS9/F167).

### Generation of consensus recommendations

The working group held two in-person forums for in-depth discussion. Initial definitions of tophus erosion severity and clinical grades were drafted based on clinical, surgical, and radiological data. Subsequently, a clinical recommendation framework was formulated (Supplemental Digital Content 5–6, available at: http://links.lww.com/JS9/F167), with multidisciplinary experts providing feedback. Two anonymous Delphi voting rounds were conducted: the first round (*n* = 50) gathered initial feedback; following revisions, the second round (*n* = 74) was conducted. Voting employed a 1–9 scale (“strongly disagree” to “strongly agree”), with responses categorized as “disapproval” (1–3), “uncertainty” (4–6), or “consensus” (7–9). Consensus was defined as ≥85% agreement.

Each recommendation was categorized as “strong” or “conditional” based on a comprehensive evaluation of benefit-risk balance and patient values. “Strong” indicated a clear advantage, whereas “conditional” reflected less definitive trade-offs.

All working group members reviewed and approved the grading definitions, recommendations, and final manuscript and accepted collective responsibility for its content. Four external auditors conducted an additional review, and CMDA approved the final version.

Two patients with tophi were interviewed anonymously to incorporate patient perspectives into the consensus recommendations. Their feedback was evaluated, and select suggestions were adopted (Supplemental Digital Content 7, available at: http://links.lww.com/JS9/F167).

## Results

**Figure 1.**
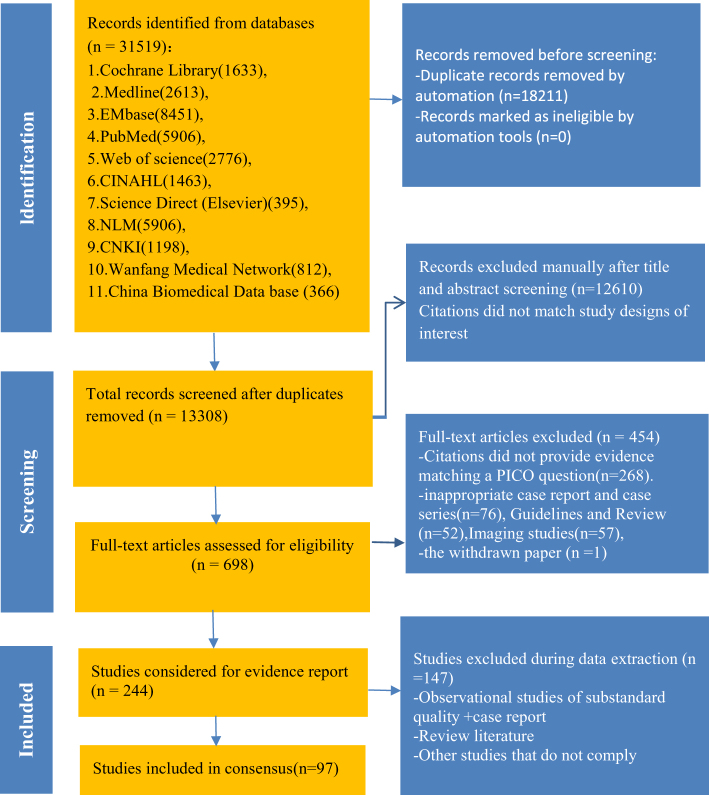
Flowchart of literature selection process(from inception until May 2025).

After screening titles and abstracts, 698 full-texts were reviewed for eligibility; 244 papers met the PICO criteria, and 97 were selected for evidence assessment and citation (Fig. 1).

**Figure 2.**
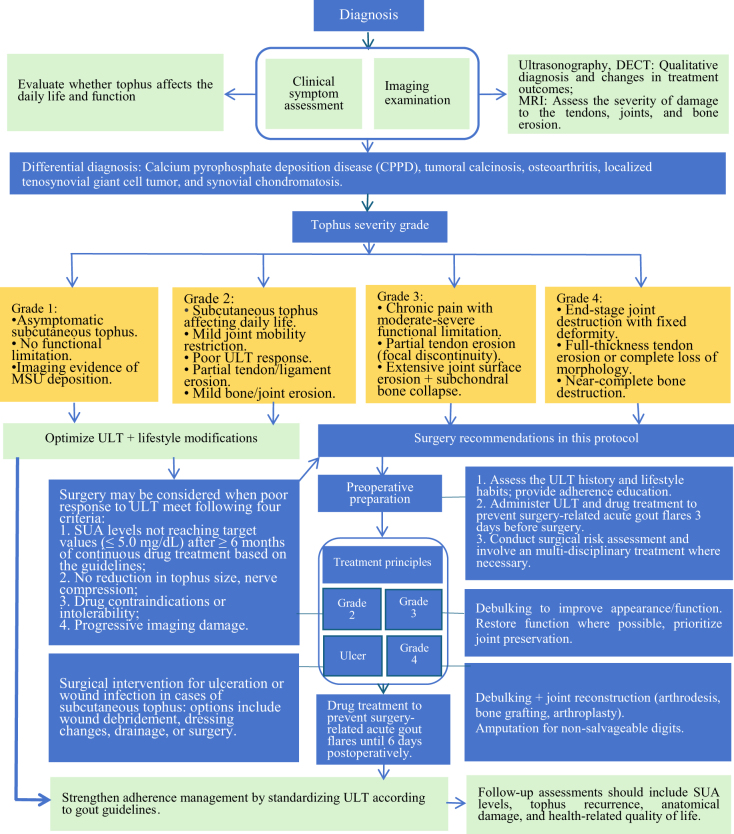
Surgical management algorithm based on grading.

Grading criteria for tophus erosion damage (grades 1–4; Table 1) and 14 consensus surgical recommendations were established: two, four, and eight for tophus erosion severity diagnosis, perioperative management, and surgical indications and techniques, respectively. Table 2 provides the strength and evidence level supporting each recommendation, and Fig. 2 illustrates the proposed treatment algorithm.

**Table 1:** Tophus erosion severity grading and treatment principles


Grade	Key features	Management strategies	Clinical notes
Grade 1	Asymptomatic subcutaneous tophus. No functional limitation. Imaging evidence of MSU deposition.	Optimize ULT + lifestyle modifications	Common in nonarticular regions and early chronic tophaceous gout.
Grade 2	Subcutaneous tophus affecting daily life. Mild joint mobility restriction. Poor ULT response. Partial tendon/ligament erosion. Mild bone/joint erosion.	Aggressive ULT + adherence education. Consider surgical debridement for tophus removal, especially combined with nerve compression, infection, or ulcer.	Surgical indication criteria:
			① Nonadherence to guidelines of undergoing ULT beyond 6 months(SUA >5.0 mg/dL)
			② No reduction in tophus size.
			③ Drugs contraindications/intolerance,
			④ Progressive imaging damage.
Grade 3	Chronic pain with moderate-severe functional limitation. Partial tendon erosion (focal discontinuity). Extensive joint surface erosion + subchondral bone collapse.	Debulking to improve appearance/function. Restore function where possible, prioritize joint preservation. Postoperative rehabilitation.	Near-irreversible damage requiring intervention.
Grade 4	End-stage joint destruction with fixed deformity. Full-thickness tendon erosion or complete loss of morphology. Near-complete bone destruction (e.g., pathologic fracture).	Debulking + joint reconstruction (arthrodesis, bone grafting, arthroplasty). Amputation for nonsalvageable digits.	Require comprehensive risk-benefit analysis: Comorbidity assessment Discussion of secondary damage risks

**Table 2:** Overview of the recommendations

Overview of the recommendations		Strong recommendation	Conditional recommendation
**Diagnosis**	Recommendation 1: It is strongly recommended that clinicians evaluate each patient’s condition based on the consensus grading criteria and treatment principles for tophus erosion damage (Table 1) and formulate diagnosis and treatment protocols accordingly (expert evidence).		
	Recommendation 2: It is strongly recommended that clinicians assess tophus erosion severity via radiological examinations. Ultrasonography and dual-energy computed tomography (DECT) can be considered for quantitative diagnosis and treatment outcome assessment, and magnetic resonance imaging (MRI) can be used to assess tophus erosion damage severity (evidence level: 2b).		
**Perioperative perio management**	Recommendation 3: It is strongly recommended that surgeons assess the history of urate-lowering therapy (ULT) and lifestyle habits of patients with tophi and provide targeted education to improve adherence to gout and tophus treatment (evidence level: 2a).		
	Recommendation 4: It is conditionally recommended that clinicians strengthen postoperative follow-up, standardize ULT and rehabilitation, and use SUA levels, tophus recurrence, and functional outcomes as primary measures for evaluating surgical treatment (expert evidence).		
	Recommendation 5: It is strongly recommended that clinicians administer perioperative drug treatment to patients with tophi to prevent surgery-related acute gout flares, provided no contraindications exist. Rheumatology consultation should be sought for personalized regimens when needed, and surgery should be avoided during acute flares (evidence level: 2b).		
	Recommendation 6: It is strongly recommended that a multidisciplinary team assess surgery-related risks for patients with tophi and certain comorbid diseases (evidence level: 1b).		
**Surgical indications and intervention methods**	Recommendation 7: It is strongly recommended that surgeons implement surgical intervention for tophi that are ulcerated, at risk of rupture, or infected. Interventions may include debridement, regular dressing changes, drainage, or advanced surgical procedures (evidence level: 1b).		
	Recommendation 8: It is strongly recommended that clinicians formulate surgical regimens based on the tophus erosion grade and treatment principles for tophi that have eroded the tendons, ligaments, joints, or bone, resulting in joint dysfunction and impaired daily activities (evidence level: 3a).		
	Recommendation 9: Surgery for tophi should not be solely based on cosmetic considerations. Recommendation 8 should serve as a reference to determine whether a tophus affects daily activities or functional capabilities (evidence level: 5).		
	Recommendation 10: It is strongly recommended that surgeons conduct surgical treatment in cases of debilitating nerve compression symptoms (persistent numbness, pain, or hypoesthesia) or when ultrasonography or electromyography indicates moderate nerve injury to prevent irreversible nerve damage (evidence level: 2b).		
	Recommendation 11: It is conditionally recommended that surgeons implement surgical intervention for tophi that fail to shrink after ULT when radiological evaluation indicates significant tissue damage. Preintervention, the ULT response and patient adherence should be assessed, and surgery options should be discussed with the patient to prevent further tissue erosion. Poor treatment response may be defined according to tophus severity grading and treatment principles (e.g., grade 2 as outlined in Table 1) (evidence level: 1b).		
	Recommendation 12: It is conditionally recommended that surgeons employ open surgery as the first-line treatment for patients with subcutaneous tophi that indicate surgery (evidence level: 3b).		
	Recommendation 13: It is conditionally recommended that surgeons consider arthroscopic surgery for intrajoint tophus shaving as an option under feasible conditions when a skilled surgeon performs it. Feasibility should be assessed based on accessibility, costs, and patient preferences (evidence level: 3b).		
	Recommendation 14: It is conditionally recommended that surgeons utilize endoscopic resection and intralesional shaving techniques for the removal of subcutaneous tophi once they have undergone softening and liquefaction. Feasibility should be assessed based on accessibility, costs, and patient preferences (evidence level: 4).		

**Recommendation 1**: It is strongly recommended that clinicians evaluate each patient’s condition based on the consensus grading criteria and treatment principles for tophus erosion damage (Table 1) and formulate diagnosis and treatment protocols accordingly (expert evidence).

No corresponding evidence was identified in the literature. Given that tophus invasion grading can help personalize treatment and management strategies – and considering the importance of postoperative assessment – the working group proposed the concept of tophus erosion damage grading, which, along with the corresponding surgical principles, was defined based on clinical features and tendon, joint, and bone damage. Agreement on the grading system was achieved through two Delphi voting rounds (≥90% agreement).

**Recommendation 2**: It is strongly recommended that clinicians assess tophus erosion severity via radiological examinations. Ultrasonography and dual-energy computed tomography (DECT) can be considered for quantitative diagnosis and treatment outcome assessment, and magnetic resonance imaging (MRI) can be used to assess tophus erosion damage severity (evidence level: 2b).

Despite being accurate, noninvasive, and readily accessible for tophus screening^[21]^, ultrasonography has lower sensitivity compared to MRI and DECT^[22,23]^, especially in cases of extra-articular uric acid deposition^[23]^. Notably, DECT helps precisely measure tophus volume and extent of crystal deposition^[24,25]^ and enables accurate evaluation of therapeutic response to tophus dissolution^[26]^. MRI provides detailed visualization of bone marrow and the soft tissues surrounding joints, ligaments, and joint cartilage^[27]^, facilitating quicker differentiation of intra-articular masses from nongouty arthritis^[28–30]^. Radiography has lower sensitivity and specificity for capturing bone erosion^[22]^. Given these strengths and limitations of each imaging technique, imaging modalities should be selected based on the diagnostic purpose.

**Recommendation 3:** It is strongly recommended that surgeons assess the history of urate-lowering therapy (ULT) and lifestyle habits of patients with tophi and provide targeted education to improve adherence to gout and tophus treatment (evidence level: 2a).

Among chronic diseases, gout is associated with the lowest level of patient adherence to treatment, with <50% of patients adhering to treatment^[17,31]^. Notably, most patients undergoing tophus surgery have nonstandardized ULT implementation^[32]^, poor adherence, and inadequate dietary control. Thus, surgeons must recognize “nonadherence as a syndrome” and anticipate poor postoperative compliance. High-quality evidence suggests that lifestyle modifications effectively help reduce serum uric acid (SUA) levels and monosodium urate deposition^[33]^. Therefore, emphasizing the importance of postoperative ULT, regular follow-ups, and adherence to a healthy, balanced diet is essential in such cases^[13,34]^.

**Recommendation 4**: It is conditionally recommended that clinicians strengthen postoperative follow-up, standardize ULT and rehabilitation, and use SUA levels, tophus recurrence, and functional outcomes as primary measures for evaluating surgical treatment (expert evidence).

Currently, specific evaluation tools to assess the effect of tophus surgery are unavailable. Lower SUA levels expedite tophus dissolution^[35]^. Maintaining SUA levels <5.0 mg/dL reduces tophus size and quantity while alleviating symptoms^[35,36]^. The working group recommends assessing SUA levels preoperatively, immediately postoperatively, and during follow-ups. Furthermore, the surgical site should be radiologically evaluated at least once, with functional assessments conducted 6–12 months postsurgery. Chronic gout outcome measures may be referenced^[37]^. Notably, surgical site pain and gout flare incidence, joint mobility range, tendon strength and function, surgical site appearance (including the presence or absence of local skin swelling), and activities of daily living scales such as the Health Assessment Questionnaire (HAQ) or the HAQ Disability Index could effectively help assess physical function in patients with gout^[38]^.

**Recommendation 5:** It is strongly recommended that clinicians administer perioperative drug treatment to patients with tophi to prevent surgery-related acute gout flares, provided no contraindications exist. Rheumatology consultation should be sought for personalized regimens when needed, and surgery should be avoided during acute flares (evidence level: 2b).

The incidence of postoperative acute gout flares in patients with a history of gouty arthritis is 17–40.3%^[7]^. Surgery-induced flares may result from significant SUA fluctuations, tissue hypoxia, and systemic inflammatory responses rather than from absolute initial SUA levels^[39,40]^. Key risk factors include drug treatment, smoking, decreased preoperative hemoglobin levels, and a substantial decrease in hemoglobin levels 1 day postsurgery^[41,42]^. Fluctuating SUA levels may partially dissolve preexisting tophi and activate proinflammatory factors, thereby increasing the risk of acute gout flares^[43]^. Preoperative prophylactic colchicine or nonsteroidal antiinflammatory drugs effectively prevent postoperative gout flares^[7,13,40,41,44]^. Initiating dosing 3 days preoperatively and continuing for 6 days postoperatively is recommended^[43,45–47]^.

**Recommendation 6:** It is strongly recommended that a multidisciplinary team assess surgery-related risks for patients with tophi and certain comorbid diseases (evidence level: 1b).

Patients with gout have an increased risk of cardiovascular and chronic kidney disease^[48,49]^, with higher all-cause mortality in those with hyperuricemia, gout, peripheral vascular disease, myocardial infarction, and heart failure^[8]^. Gout is also a risk factor for ischemic stroke^[50]^, and patients with tophi and renal impairment experience significantly prolonged hospitalization^[11]^. The working group recognizes that older patients with gout and multiple comorbidities (such as chronic kidney disease, cardiovascular disease, obesity, or diabetes mellitus) are at heightened risk of developing surgical complications. Therefore, preoperative risk assessment is essential.

**Recommendation 7:** It is strongly recommended that surgeons implement surgical intervention for tophi that are ulcerated, at risk of rupture, or infected. Interventions may include debridement, regular dressing changes, drainage, or advanced surgical procedures (evidence level: 1b).

Tophus ulceration at joints and weight-bearing sites is prone to delayed healing^[51]^. Healing may take >3 years in some cases, and the QoL scores of these patients are worse than those of patients with chronic lower limb ulceration^[52]^. Approximately 67% of patients with delayed surgical site healing experienced tophus ulceration or infection peroperatively^[18]^, often prolonging hospitalization^[11]^. Therefore, strengthened surveillance of tophus ulceration and evaluation of wound care strategies are essential to reducing ulcer incidence^[51]^. Small, superficial ulcers may be managed locally (e.g., using a 3% citric acid gel) to promote granulation and healing^[53,54]^. Fenestration and drainage, dressing changes, or intralesion suction may be effective for softened or fluctuant tophi^[11]^. Surgical approaches, such as skin grafting and flap repair^[55]^, can accelerate healing, prevent superinfections, and reduce the formation of new ulcers^[53,56]^.

Combining surgical or ultrasound-assisted wound debridement with negative-pressure treatment for tophus ulceration facilitates postoperative wound drainage, reduces the risk of infection, and shortens hospitalization^[57]^. Arthroscopic or open debridement combined with intermittent negative-pressure treatment and irrigation has also proven effective for treating intraarticular tophi and severe comorbid septic arthritis^[58]^.

**Recommendation 8:** It is strongly recommended that clinicians formulate surgical regimens based on the tophus erosion grade and treatment principles for tophi that have eroded the tendons, ligaments, joints, or bone, resulting in joint dysfunction and impaired daily activities (evidence level: 3a).

Tophi contribute to muscle loss and disuse atrophy^[59]^, along with other significant effects on daily activities and QoL^[60]^. Surgical resection of joint tophi can prevent further destruction of bone, joints, and soft tissues, mitigate severe deformities, maintain joint stability, and improve joint function^[61]^. Surgery is also effective in preventing disease progression^[62]^. Most postoperative complications are minor^[32,63]^, with delayed wound healing being the most common. Observational studies have demonstrated that pain symptoms, activity, functionality, and patient satisfaction improve during follow-up, with no recurrence reported in the absence of skin necrosis postoperatively^[61,62,64]^. Thus, meticulous surgical management is warranted to minimize complications.

**Recommendation 9:** Surgery for tophi should not be solely based on cosmetic considerations. Recommendation 8 should serve as a reference to determine whether a tophus affects daily activities or functional capabilities (evidence level: 5).

Some surgeons consider cosmetic needs as an indication for surgery^[11,62,65]^. However, among the eight available guidelines and consensus guidelines on surgical indications, only one cited cosmetics as a surgical indication and emphasized that surgery should be conducted after drug treatment^[66]^. Given the cultural preferences and customs in China, patients typically opt for surgery based on functional impairments rather than cosmetic concerns. Furthermore, all surgeries are associated with potential risks.

**Recommendation 10**: It is strongly recommended that surgeons conduct surgical treatment in cases of debilitating nerve compression symptoms (persistent numbness, pain, or hypoesthesia) or when ultrasonography or electromyography indicates moderate nerve injury to prevent irreversible nerve damage (evidence level: 2b).

Tophus invasiveness increases compression of local nerves,^[67–69]^, blood vessels, and tendons, causing intractable pain; early intervention can prevent irreversible nerve damage^[68,69]^. Clinical signs and symptoms, as well as ultrasonography findings, can effectively help determine nerve compression severity^[70,71]^. Ultrasonography is a simple, reliable, and convenient diagnostic tool for such cases^[72]^. Surgical decompression is beneficial for alleviating tophus-related nerve compression symptoms, and epineural release may be considered in cases of severe compression^[47]^. Liu, *et al*^[69]^ reported that superficial flexor tendon resection to reduce carpal tunnel content did not impair ipsilateral finger activity postoperatively^[69]^. Conversely, Onuma, *et al*^[73]^ advocated for observation and continued ULT in cases of mild numbness^[73]^.

**Recommendation 11**: It is conditionally recommended that surgeons implement surgical intervention for tophi that fail to shrink after ULT when radiological evaluation indicates significant tissue damage. Preintervention, the ULT response and patient adherence should be assessed, and surgery options should be discussed with the patient to prevent further tissue erosion. Poor treatment response may be defined according to tophus severity grading and treatment principles (e.g., grade 2 as outlined in Table 1) (evidence level: 1b).

SUA levels generally decrease to ~5.0 mg/dL after ≥6 months of ULT, leading to gradual tophus dissolution^[36]^. However, approximately 5% of patients fail to meet target levels despite ULT, potentially owing to drug resistance^[9]^. Early surgical resection can prevent further erosion and damage to critical tissues and organs while reducing the risk of tophus infection or ulceration^[9,11,32,62,74]^. Surgery may also be appropriate for patients with persistent SUA levels of 5.0–5.5 mg/dl despite tolerability to treatment^[75]^. Empirical studies suggest that surgical intervention contributes to the reduced recurrence of subcutaneous tophi in situ as an added benefit^[62,64,76]^. Tophi can cause irreversible structural damage to joints and tendons^[73,77]^, which is challenging to reverse despite high-dose or combined ULT^[78]^.

Significant long-term improvements in SUA levels, mobility, and acute gout flares frequency were observed in patients undergoing arthroscopy followed by postoperative ULT than in those receiving drug monotherapy alone^[61,79–81]^. The SF-36 score was fourfold higher than that of the drug group^[81]^. Case reports have also documented rapid tophus disappearance on hands and feet following ULT and surgical resection of massive tophi on contralateral extremities^[73,82]^.

**Recommendation 12**: It is conditionally recommended that surgeons employ open surgery as the first-line treatment for patients with subcutaneous tophi that indicate surgery (evidence level: 3b).

In most studies, open resection has been the primary tophus surgery approach^[32,63,83]^. Its advantages include simplicity of required equipment, sufficient exposure under direct visualization, minimal healthy tissue injury, thorough tophus removal, and effective hemostasis and irrigation.

Sharp resection is preferred over blunt dissection to minimize local tissue damage, ischemia, and necrosis^[5,9,62]^. Tophi should be dissected under their capsules, and thin skin should be avoided to preserve dermal vasculature. For larger or complex lesions, incisions should be lengthened to fully expose the surgical field of view. Therefore, flap or skin grafting may be required for wound healing^[55]^.

Tophi should be extensively removed when tendon invasion is minimal or absent. Large extra-tendinous tophi should be debulked to correct tendon deviation, release adhesions, alleviate compression, and restore tendon mobility^[84]^. For tophi-enveloped tendons, a layer-by-layer removal along the exposed tendon is recommended. As postoperative tendon rehabilitation remains unexplored, the working group recognizes the need for postoperative adjuvant functional rehabilitation.

Arthroplasty or arthrodesis of the first metatarsophalangeal joint is suitable when tophi erode >50% of the joint cartilage. Arthrodesis offers superior outcomes in terms of pain relief, American Orthopedic Foot & Ankle Society score, and patient satisfaction scores compared with that offered by tophus resection alone^[65,79]^. Bone healing using the Ilizarov technique for first metatarsophalangeal joint tophus defects has also yielded favorable outcomes but requires specialized surgical skills, meticulous care for external fixation devices, and high patient adherence^[46]^. These evidence suggest that surgery can moderately improve small joint deformities, preserve joint function, and enhance QoL. However, surgical complexity for grade 4 tophi requires careful consideration. Severe erosion is associated with a poorer prognosis. Evidence on surgical management of severe tophus fusion, joint replacement, and large joint defect repair is currently insufficient to formulate recommendations.

**Recommendation 13**: It is conditionally recommended that surgeons consider arthroscopic surgery for intra-joint tophus shaving as an option under feasible conditions when a skilled surgeon performs it. Feasibility should be assessed based on accessibility, costs, and patient preferences (evidence level: 3b).

Arthroscopic treatment of intrajoint tophi is associated with shorter hospitalization, reduced inflammatory markers, improved pain and functional scores, fewer complications, and enhanced postoperative recovery compared with those of conventional surgery ^[85–87]^. Arthroscopy enables definitive diagnosis, comprehensive intra-joint lesion removal, and early prevention of joint cartilage destruction^[88]^.

However, limited joint space and technical difficulty restrict its use for small joints such as the metatarsophalangeal joints^[89]^. Contraindications include a history of knee surgery, severe trauma, and comorbidities such as abnormal coagulation, local skin infection of the joint, or preoperative thrombosis^[61]^.

**Recommendation 14**: It is conditionally recommended that surgeons utilize endoscopic resection and intralesional shaving techniques for the removal of subcutaneous tophi once they have undergone softening and liquefaction. Feasibility should be assessed based on accessibility, costs, and patient preferences (evidence level: 4).

Endoscopic resection allows minimally invasive tophus removal under visual guidance, thereby preventing damage to healthy tissues^[89,92]^, reducing intraoperative blood loss, and shortening surgical duration^[11,45,93]^. However, the shaving technique is performed without direct visual guidance and carries the risk of damage to neighboring blood vessels, nerves, and tendons, rendering it difficult to completely remove nonsoftened tophi. Therefore, sharp curette shaving is not recommended when the tophus involves tendons, ligaments, or joint capsules^[45]^.

Numerous tophi are a contraindication for endoscopy^[94]^. Shaving is associated with increased recurrence risk and limited improvement in appearance and range of mobility. Conversely, open surgery provides better visualization of tendons, nerves, and blood vessels, allowing complete tophus removal and improved mobility^[84]^. However, no study has compared the follow-up outcomes of these surgical methods.

## Discussion

Tophus surgery may occur more frequently in clinical practice than reported^[17]^, highlighting the urgent need for standardized surgical guidelines. Many surgeons advocate developing practice-based guidelines^[5,9,95,96]^. Accordingly, this first consensus guideline was designed to address core clinical issues in surgical tophus management by systematically evaluating 11 key questions across diagnosis, perioperative management, surgical strategies, and technique. Emphasis was placed on safety and complications to guide focused consensus development. For areas lacking sufficient evidence or clarity, this consensus calls for increased attention to standardized postoperative functional assessment (recommendation 4). Additionally, patient perspectives are fully integrated (recommendation 9), embodying the principle of patient-centered care.

This consensus adds to the existing literature and differs from previous drug-focused guidelines in several key respects. First, it represents a surgeon-led multidisciplinary initiative, in collaboration with experts from rheumatology, imaging, rehabilitation, nursing, and wound management, to formulate the first actionable technical framework for the surgical management of tophaceous gout. These guidelines introduce the first severity grading system, enabling standardized outcome comparisons and addressing the absence of lesion-specific surgical approaches for tophi that currently contribute to variable outcomes. Therefore, establishing a unified standard is the first step toward ensuring consistency and comparability in surgical interventions.

Second, while existing guidelines primarily emphasize pharmacotherapy and patient education^[2,15,36]^, these recommendations emphasize evaluating anatomical and functional impairments as key treatment outcomes alongside ULT standards. This concept formally integrates surgical treatment into comprehensive gout management for the first time, shifting from a “metabolic control-only” model to a “metabolic–structural–functional” integrated management approach. This model requires close surgeon–physician collaboration, particularly in community healthcare settings.

Third, while surgery is not curative, it precedes and supports the use of ULT. Continuing education and training can help surgeons recognize surgery not as an endpoint but as part of a continuum encompassing regular follow-up, medication guidance, and supervision of patients’ functional rehabilitation management, forming a closed-loop management model of “surgical intervention–drug maintenance–functional rehabilitation.”

This study has some limitations. First, most evidence on surgical interventions for tophi is derived from retrospective or observational studies of limited quality, with few RCTs, thereby reducing the strength of recommendations and reinforcing the urgent need for expert consensus. Second, the Delphi method may exclude emerging viewpoints lacking strong evidence, and the proposed grading system requires clinical validation. Broader implementation and promotion of this consensus would require the development of additional evaluation criteria and tools, dissemination at academic conferences, gathering clinician feedback via questionnaires, and expanded international collaboration to update the recommendations. Third, health economic recommendations could not be provided owing to insufficient evidence^[97]^, including cost-effectiveness analyses of surgical interventions and long-term healthcare resource utilization. Finally, while the recommendations address common clinical situations, they do not cover all cases, and individualized treatment remains essential.

Surgical management of tophi requires increased global clinical focus. Currently, few optimal surgical interventions exist. Future research priorities include investigating global epidemiology to establish a disease burden database; developing cost-effectiveness models incorporating medical and societal costs and quality-adjusted life years to support healthcare policy decisions^[98]^; conducting high-quality RCTs comparing long-term surgical versus intensive medical management; validating the proposed severity grading system’s clinical and cross-cultural utility through international multicenter cohort studies; and developing patient-reported outcome measures addressing QoL for patients with gouty tophi.

In conclusion, despite limitations in evidence strength, this first surgically led, multidisciplinary consensus on tophus management leverages clinical expertise and a rigorous development process to provide practical guidance for complex cases. Its primary innovation lies in integrating surgery into gout management and introducing a grading system for tophus erosion severity, enabling standardized assessment and evidence-based treatment. This structured framework addresses key gaps by offering 14 actionable recommendations across perioperative care, surgical indications, and techniques. These redefine surgery as a core component of comprehensive gout care and shift the model from drug-therapy-only to a metabolic–structural–functional approach that incorporates surgery with drug maintenance and rehabilitation. The consensus also outlines essential learning points for clinicians, including appropriate imaging (DECT/MRI), criteria for surgical intervention (including nerve compression and ulceration), and the importance of postoperative adherence and functional recovery. As high-quality research emerges, this consensus will evolve to further standardize tophus management. The development group will continue monitoring advancements and updating recommendations to ensure sustained evidence-based guidance.

## Members of the CMDA consensus panel

**Table:** Members of the CMDA Consensus Panel

No.	Name	Affiliation
1	Prof. Yonghua Sun	Ex-Chairman of Chinese Burn Association, Consultant of Chinese Medical Doctor Association Wound Repair Committee, Beijing Jishuitan Hospital, Beijing.
2	Dahai Hu, MD	Ex-Chairman of Chinese Burn Association, Department of Burns and Cutaneous Surgery, Xijing Hospital, Air Force Medical University, Xi’an, Shaanxi, China.
3	Guangping Liang, MD	Burns Research Institute, Southwest Hospital of Army Medical University, Chongqing.
4	Jiaping Zhang MD	Standing committee member, Chinese Medical Doctor Association Wound Repair Committee, Plastic Surgery Department, Southwest Hospital of Army Medical University, Chongqing.
5	Yi Zhang, MD	Chairman-elect, Chinese Medical Doctor Association Wound Repair Committee, Department of Burn and Plastic Surgery and Wound Repair, Affiliated Hospital of Nantong University, Nantong, Jiangsu.
6	Prof. Xiaolong Liu, Chief Physician	Vice Chairman, Chinese Medical Doctor Association Wound Repair Committee, Department of Burn and Plastic Surgery and Wound Repair, People’s Hospital of Xinjiang Uygur Autonomous Region, Urumqi, Xinjiang.
7	Prof. Binghui Li, Chief Physician	Vice Chairman, Chinese Medical Doctor Association Wound Repair Committee, Department of Wound Repair, Liyuan Hospital of Tongji Medical College of Huazhong University of Science & Technology, Wuhan, Hubei.
8	Shuhua Liu, MD	Standing committee member, Chinese Medical Doctor Association Wound Repair Committee, Department of Burn and Plastic Surgery, Wuhan Third Hospital, Wuhan, Hubei.
9	Pihong Zhang, MD	Standing committee member, Chinese Medical Doctor Association Wound Repair Committee, Department of Burn and Plastic Surgery and Wound Repair, Xiangya Hospital of Central South University, Changsha, Hunan.
10	Junli Zhou, MD	Standing committee member, Chinese Medical Doctor Association Wound Repair Committee, Department of Burn and Plastic Surgery and Wound Repair, Eighth Affiliated Hospital of Southern Medical University, Guangzhou, Guangdong.
11	Rungong Yang, MD	Standing committee member, Chinese Medical Doctor Association Wound Repair Committee, Department of Wound Repair, 301 Hospital, Beijing.
12	Yiwen Niu, MD	Standing committee member, Chinese Medical Doctor Association Wound Repair Committee, Department of Wound Repair, Ruijin Hospital Affiliated to Shanghai Jiaotong University School of Medicine, Shanghai.
13	Yibing Wang, MD	Standing committee member, Chinese Medical Doctor Association Wound Repair Committee, Department of Wound Repair, Shandong Provincial Qianfoshan Hospital, Jinan, Shandong.
14	Zhaohong Chen, MD	Standing committee member, Chinese Medical Doctor Association Wound Repair Committee, Department of Burn and Plastic Surgery and Wound Repair, Fujian Medical University Union Hospital, Fuzhou, Fujian.
15	Guoxian Chen, MD	Standing committee member, Chinese Medical Doctor Association Wound Repair Committee, Department of Burn and Plastic Surgery and Wound Repair, Second Affiliated Hospital of Zhejiang University School of Medicine, Hangzhou, Zhejiang.
16	Biao Cheng, MD	Standing committee member, Chinese Medical Doctor Association Wound Repair Committee, Department of Plastic Surgery, PLA Southern Theater Command General Hospital, Guangzhou, Guangdong.
17	Ke Tao, MD	Department of Wound Repair, First Affiliated Hospital of Wenzhou Medical University, Wenzhou, Zhejiang.
18	Guang Feng, MD	Department of Wound Repair, Peking University Shougang Hospital, Beijing.
19	Jing Li, MD	Department of Burns, Plastic Surgery, and Wound Repair, Tangdu Hospital, Xi’an, Shanxi.
20	Juntao Cheng, MD	Department of Burns, Plastic Surgery, and Wound Repair, Quanzhou First Hospital Affiliated to Fujian Medical University, Quanzhou, Fujian.
21	Hailin Xu, MD	Standing committee member, Chinese Medical Doctor Association Wound Repair Committee, Department of Orthopedics, Peking University First Hospital, Beijing.
22	Qikai Hua, MD	Department of Orthopedics, First Affiliated Hospital of Guangxi Medical University, Nanning, Guangxi.
23	Gang Du, MD	Department of Orthopedics, First Affiliated Hospital of Guangxi Medical University, Nanning, Guangxi.
24	Jian Li, MD	Sports Medicine Center, West China University Hospital, Sichuan University, Chengdu, Sichuan.
25	Prof. Minghuan Zhang, Chief Physician	Department of Orthopedics, Wuhan Third People’s Hospital, Wuhan, Hubei.
26	Prof. Wen Mao, Chief Physician	Orthopedics Department, Wuhan Third People’s Hospital, Wuhan, Hubei.
27	Xiang Gao, MD	Department of Bone and Joint Surgery, The Second Affiliated Hospital of Zhejiang University School of Medicine, Hangzhou, Zhejiang.
28	Cong Wang, MD	Department of Bone and Joint Surgery, The Second Affiliated Hospital of Zhejiang University School of Medicine, Hangzhou, Zhejiang.
29	Yong Ding, MD	Department of Orthopedics, Xi’anHonghui Hospital, Xi’an, Shaanxi.
30	Prof. Jing Chen, Chief Physician	Department of Bone and Joint Surgery, Beijing Aerospace General Hospital, Beijing.
31	Yueshu Wang, MD	Department of Hand Surgery, China-Japan Union Hospital of Jilin University, Changchun, Jilin.
32	Min Chen, MD	Department of Orthopedics, Fujian Medical University Union Hospital, Fuzhou, Fujian.
33	Wenge Liu, MD	Department of Orthopedics, Fujian Medical University Union Hospital, Fuzhou, Fujian.
34	Fen Li, MD	Department of Rheumatology and Immunology, Second Xiangya Hospital of Central South University, Changsha, Hunan.
35	Zhichun Liu, MD	Department of Rheumatology and Immunology, Second Affiliated Hospital of Soochow University, Suzhou, Jiangsu.
36	Ling Fang, MD	Department of Rheumatology and Immunology, Southern University of Science and Technology, Shenzhen, Guangdong.
37	Junna Ye, MD	Department of Rheumatology and Immunology, Ruijin Hospital Affiliated to Shanghai Jiaotong University School of Medicine, Shanghai.
38	Huaxiang Wu, MD	Department of Rheumatology and Immunology, The Second Affiliated Hospital of Zhejiang University School of Medicine, Hangzhou, Zhejiang.
39	Lina Zhou MD	Department of Rheumatology and Immunology, Southwest Hospital of the Third Military Medical University, Chongqing.
40	Prof. Jinying Lin, Chief Physician	Department of Rheumatology and Immunology, People’s Hospital of Guangxi Zhuang Autonomous Region, Nanning, Guangxi.
41	Ling Zhao MD	Department of Rheumatology and Immunology, First Bethune Hospital of Jilin University. Changchun, Jilin.
42	Xinwang Duan, MD	Department of Rheumatology and Immunology, Second Affiliated Hospital of Nanchang University Ranking, Nanchang, Jiangxi.
43	Xiangxiong Zheng, MD	Department of Rheumatology, Fujian Medical University Union Hospital, Fuzhou, Fujian.
44	Risheng Yu, MD	Medicine Imaging Department, The Second Affiliated Hospital of Zhejiang University School of Medicine, Hangzhou, Zhejiang.
45	Huiping Shi, MD	Medical Imaging Department, Air Force Medical Center, Beijing.
46	Xin Sun, MD	Medical Imaging Department, Xiehe Hospital, Tongji Medical College,Huazhong University of Scienceand Technology
47	Yujin Wang MD	Medical Imaging Department, Tongji Hospital,Tongji Medical College, Huazhong University of Science and Technology.
48	Shouping Dai, MD	Medical Imaging Department, Fifth Affiliated Hospital of Shandong University, Linyi, Shandong.
49	Yunjing Xue, MD	Medicine Imaging Department, Fujian Medical University Union Hospital, Fuzhou, Fujian.
50	Yumin Li, MD	Medicine Imaging Department, Wuhan Union Hospital, Wuhan, Hubei.
51	Aihua Chen, Chief nurse	Wound Ostomy Center, Wenzhou Heping International Hospital, Wenzhou, Zhejiang.
52	Liqun Zhu, Chief nurse	Evidence Application Base, Affiliated Hospital of Jiangsu University, Zhenjiang, Jiangsu.
53	Qin Zhou, Chief nurse	Department of Burns and Cutaneous Surgery, Xijing Hospital, Air Force Medical University, Xi’an, Shaanxi, China.
54	Yunmin Cai, Chief nurse	Wound Center, Jinshan Hospital of Fudan University, Shanghai.
55	Huwen Wu, MMed	Department of Wound Repair, First Affiliated Hospital of Hainan Medical University, Haikou, Hainan.
56	Prof. Zongyu Li	Department of wound Repair Surgery, Harbin Fifth People’s Hospital, Heilongjing, China.
57	Prof. Xingang Zhen MMed	Department of Bone and Joint Surgery, Harbin Fifth People’s Hospital, Heilongjing, China.
58	Yi Liu MD	Department of Rheumatology and Immunology, West China Hospital, Sichuan University.Chengdu, Sichuan.
59	Prof. Yiqun Sun Mph	Plastic and Burn Department, Western Central Hospital, Western Central Hospital, Danzhou, Hainan, China.

## Data Availability

The data used to support the findings of this study can be released upon request.

## Research registration unique identifying number (UIN)

Unique Identifying number or registration ID: Researchregistry11019 http://www.researchregistry.com
